# Antimicrobial susceptibility and resistance mechanisms to antipseudomonal β-lactams in *Pseudomonas aeruginosa* isolates from blood

**DOI:** 10.1128/spectrum.02790-24

**Published:** 2025-03-31

**Authors:** Mika Murata, Kosuke Kosai, Fujiko Mitsumoto-Kaseida, Norihito Kaku, Hiroo Hasegawa, Koichi Izumikawa, Hiroshi Mukae, Katsunori Yanagihara

**Affiliations:** 1 Department of Laboratory Medicine, Nagasaki University Hospital 88380, Nagasaki, Nagasaki Prefecture, Japan; 2 Department of Laboratory Medicine, Nagasaki University Graduate School of Biomedical Sciences 723477, Nagasaki, Nagasaki Prefecture, Japan; 3 Department of Infectious Diseases, Nagasaki University Graduate School of Biomedical Sciences 200674, Nagasaki, Nagasaki Prefecture, Japan; 4 Department of Respiratory Medicine, Nagasaki University Graduate School of Biomedical Sciences 723481, Nagasaki, Nagasaki Prefecture, Japan; JMI Laboratories, North Liberty, Iowa, USA

**Keywords:** bloodstream infection, resistance to antipseudomonal β-lactams, gene expression, ceftolozane/tazobactam, cefiderocol

## Abstract

Bloodstream infection caused by *Pseudomonas aeruginosa* with antimicrobial resistance can be difficult to treat. Herein, we investigated antimicrobial susceptibility to major antipseudomonal β-lactams and analyzed the relationship between resistance mechanisms and susceptibilities in *P. aeruginosa* isolates from blood. A total of 97 isolates possessing no carbapenemase gene were included in this study. The susceptibility rates to piperacillin, piperacillin/tazobactam, ceftazidime, and cefepime were in the 80% range, while 74.2% and 80.4% of the isolates showed susceptibility to imipenem and meropenem, respectively. None of the isolates showed resistance to ceftolozane/tazobactam or cefiderocol, and only one isolate each showed intermediate resistance (susceptibility rate, 99.0% each). The susceptibility rate was lowest for aztreonam (71.1%). The most prevalent susceptibility pattern was susceptible to all agents (56 isolates), followed by not susceptible only to aztreonam (nine isolates) or imipenem (seven isolates), and susceptible only to both ceftolozane/tazobactam and cefiderocol (six isolates). Multivariate logistic regression analysis revealed significant correlations between decreased *oprD* expression and decreased susceptibility to carbapenems; between increased *ampC* expression and non-susceptibility to piperacillin, piperacillin/tazobactam, and ceftazidime; and between increased *mexA* expression and decreased susceptibility to piperacillin, piperacillin/tazobactam, cefepime, aztreonam, and meropenem. These findings highlight the correlations between the expression of AmpC β-lactamase, OprD porin, and efflux pumps and non-susceptibility to β-lactams in *P. aeruginosa* isolates from blood. In contrast to traditional β-lactams, the stability of ceftolozane/tazobactam and cefiderocol against the resistance mechanisms was confirmed.

**IMPORTANCE:**

*Pseudomonas aeruginosa* is a significant pathogen in hospital-acquired bloodstream infection (BSI) whose treatment requires the appropriate antimicrobial selection in consideration of antimicrobial resistance and resistance mechanisms. Although antipseudomonal β-lactams are key antimicrobial agents, several molecular mechanisms, including β-lactamases, OprD porin, and efflux pumps, are known to be intricately involved in β-lactam resistance. This study evaluated susceptibility profiles of *P. aeruginosa* to antipseudomonal β-lactams and revealed the correlations between susceptibility and the expression of genes underlying the relevant resistance mechanisms. The results demonstrate the potent *in vitro* activity of two new agents, ceftolozane/tazobactam and cefiderocol, against *P. aeruginosa* showing resistance to traditional antipseudomonal β-lactams. This study provides insights into the mechanisms underlying antipseudomonal β-lactam resistance in *P. aeruginosa*, highlighting the potential of ceftolozane/tazobactam and cefiderocol for use against *P. aeruginosa* BSI.

## INTRODUCTION

Bloodstream infection (BSI) caused by *Pseudomonas aeruginosa* is a serious condition associated with a high mortality rate (1, 2). Antimicrobial resistance, including carbapenem resistance in *P. aeruginosa*, is associated with excessive all-cause mortality in cases of BSI (3). Antipseudomonal β-lactams, including piperacillin/tazobactam, ceftazidime, cefepime, and carbapenems, are traditional therapeutic options against *P. aeruginosa* infections (4, 5). Recent studies have reported resistance rates ranging from 8.9% to 17.9% in Japan and 6% to 11% in the United States to these agents for *P. aeruginosa* isolates obtained from patients with BSI (2, 6). Furthermore, several new β-lactams, including ceftolozane/tazobactam and cefiderocol, show promise for use as treatment options for infections caused by *P. aeruginosa* with antimicrobial resistance (4, 5). Ceftolozane is an antipseudomonal cephalosporin with improved stability against the chromosomal AmpC produced by *P. aeruginosa* (7, 8). Cefiderocol is a synthetic conjugate composed of a cephalosporin and a catechol-type siderophore that chelates iron, facilitating entry to bacterial cells via active iron transporters (9, 10). We recently reported that the susceptibility rate to ceftolozane/tazobactam was 92.9% in *P. aeruginosa* isolates obtained from patients with BSI at three Japanese university hospitals (2). In another study conducted in Europe, the susceptibilities of non-carbapenem-resistant *P. aeruginosa* isolates from patients with BSI to ceftolozane/tazobactam and cefiderocol were found to be 96.7% and 99.7%, respectively, whereas those of the carbapenem-resistant isolates were 37.8% and 97.8%, respectively (10).

Resistance to β-lactams is mediated by several molecular mechanisms, including β-lactamases, OprD porin, and efflux pumps (11–13). Although these resistance mechanisms to traditional β-lactams are generally recognized in *P. aeruginosa*, a detailed epidemiology and their precise characteristics have yet to be comprehensively investigated, especially in our region. In addition, the way these resistance mechanisms affect the susceptibility of *P. aeruginosa* to newer agents remains unclear.

In this study, we investigated the antimicrobial susceptibilities of *P. aeruginosa* to major antipseudomonal β-lactams, including newer agents, and analyzed the relationship between resistance mechanisms and susceptibilities in *P. aeruginosa* isolates from the blood of patients in our hospital.

## MATERIALS AND METHODS

### Bacterial isolates and antimicrobial susceptibility testing

We used *P. aeruginosa* isolates from blood samples between January 2013 and June 2020 at the Nagasaki University Hospital. Bacteria were cultured in LB broth with shaking at 35℃ ± 2°C overnight. No duplicate isolates from a single patient were included. Antimicrobial susceptibility was examined using BD Phoenix M50 (Becton Dickinson) according to the manufacturer’s instructions. Antimicrobial susceptibility to cefiderocol was tested using a minimal inhibitory concentration (MIC) plate according to the manufacturer’s instructions. Briefly, serial dilutions of cefiderocol were prepared in sterile saline. The isolate was suspended in iron-depleted cation-adjusted Mueller Hinton Broth (ID-CAMHB), adjusted to a 0.5 McFarland standard, and diluted 10-fold using ID-CAMHB. Next, 5 µL each of the isolate suspension and drug solution was inoculated into 90 µL of ID-CAMHB on the plate. The MIC plate prepared was incubated at 35°C ± 2°C for 16–20 h. Antimicrobial susceptibility was determined according to the Clinical and Laboratory Standards Institute document M100-Ed34.

### Detection of carbapenemase and extended-spectrum β-lactamase genes

Carbapenemase genes were examined using a multiplex PCR assay kit, the Cica Geneus Carbapenemase Genotype Detection Kit 2 (Kanto Chemical Co., Inc.) according to the manufacturer’s instructions. The kit can detect *bla*_IMP-1_ group, *bla*_IMP-6_, *bla*_VIM_ group, *bla*_NDM_ group, *bla*_OXA-48_ group, *bla*_KPC_ group, and *bla*_GES_ group carbapenemases (14). For bands suspected as nonspecific amplification, singleplex assays for *bla*_IMP_ group, *bla*_VIM_ group, and *bla*_OXA-48_ group were additionally performed using PCR with the following primers (15): *bla*_IMP_ group forward, 5′-GGAATAGAGTGGCTTAAYTCTC-3′; *bla*_IMP_ group reverse, 5′-GGTTTAAYAAAACAACCACC-3′; *bla*_VIM_ group forward, 5′-GATGGTGTTTGGTCGCATA-3′; *bla*_VIM_ group reverse, 5′-CGAATGCGCAGCACCAG-3′; *bla*_OXA-48_ group forward, 5′-GCGTGGTTAAGGATGAACAC-3′; and *bla*_OXA-48_ group reverse, 5′-CATCAAGTTCAACCCAACCG-3′. Amplification was conducted under the following conditions: 10 min at 94°C; 36 cycles of 30 s at 94°C, 40 s at 52°C, and 50 s at 72°C; and 5 min at 72°C for the final extension.

Extended-spectrum β-lactamase (ESBL) genes were examined using a multiplex PCR assay kit, the Cica Geneus ESBL Genotype Detection Kit 2 (Kanto Chemical Co., Inc.), which can detect *bla*_CTX-M-1_ group, *bla*_CTX-M-2_ group, *bla*_CTX-M-8_ group, *bla*_CTX-M-9_ group, *bla*_CTX-M-25_ group, *bla*_CTX-M chimera_, *bla*_GES_ (ESBL type), *bla*_TEM_, and *bla*_SHV_, according to the manufacturer’s instructions.

### Gene expression of OprD porin, AmpC cephalosporinase, and Mex efflux transporters

RNA was extracted using the MagMAX-96 for Microarrays Total RNA Isolation Kit (Thermo Fisher Scientific) and reverse-transcribed into cDNA using the ReverTra Ace qPCR RT Master Mix with gDNA Remover (Toyobo) according to the manufacturer’s instructions. Real-time PCR was performed with THUNDERBIRD SYBR qPCR Mix (Toyobo) using LightCycler 480 system II (Roche Diagnostics) according to the following profile: an initial incubation (1 min at 95°C), amplification (45 cycles consisting of 15 s at 95°C; 30 s at 60°C for *oprD* and *16S rRNA*/30 s at 70°C for *ampC*, *mexA*, *mexC*, *mexE*, and *mexX*; and 20 s at 72°C), and high-resolution melting analysis (1 min at 95°C, 1 min at 40°C, 1 s at 60°C, and heating up to 95°C at a rate of 0.11°C/s). The primers used are listed in Table S1 (16–18).

To generate calibration curves for quantitative analysis, PCR for each gene was performed using the AmpliTaq Gold 360 Master Mix (Thermo Fisher Scientific), and the products were purified using the QIAquick PCR Purification Kit (QIAGEN). The gene copy numbers were measured using the Quant-i PicoGreen dsDNA Assay Kits and dsDNA Reagents (Invitrogen) according to the manufacturer’s instructions. Target gene expression was normalized to *16S rRNA* (19–21) by dividing the absolute copy number of each target gene by that of each *16S rRNA* in the clinical isolates and *P. aeruginosa* PAO1 strain. The fold change was calculated by dividing the normalized target gene expression of the isolate by that of the *P. aeruginosa* PAO1 strain.

### Statistical analysis

The numerical data of normalized gene expression were compared using the Wilcoxon rank-sum test between groups (22). Univariate and multivariate analyses were performed using a nominal logistic regression model to detect correlations between resistance gene expression and non-susceptibility (intermediate and resistant) to each β-lactam. Variables with *P* values <0.2 in univariate analysis were selected and adjusted by multivariate analysis. Data were analyzed using JMP v16 (SAS Institute Inc.), and the results were considered statistically significant at *P*  <0.05.

## RESULTS

**Fig 1:**
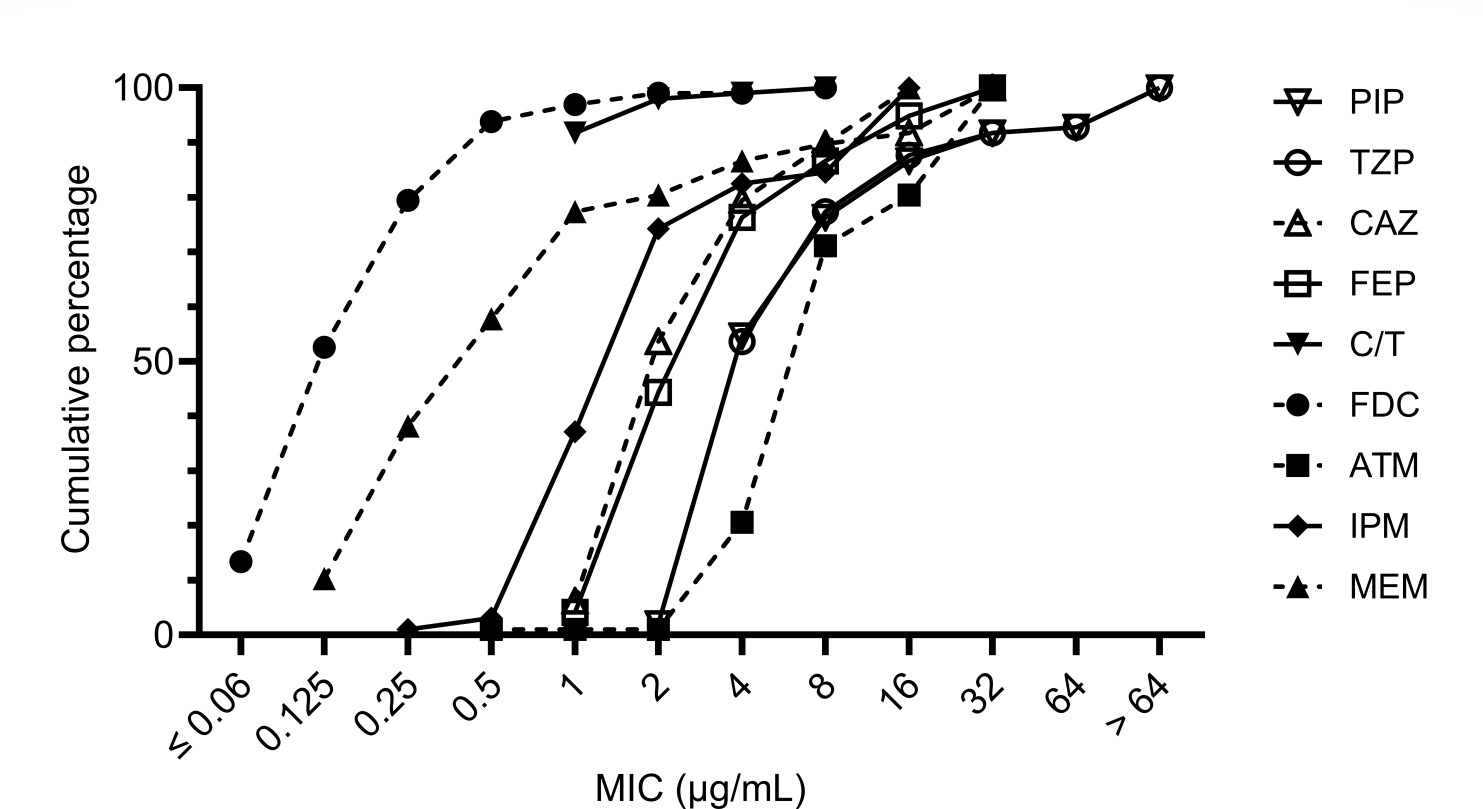
Cumulative minimal inhibitory concentration distribution in 97 *Pseudomonas aeruginosa* isolates. MIC values were categorized as follows: PIP: ≤2 µg/mL as 2 µg/mL; TZP: ≤4/4 µg/mL as 4/4 µg/mL; CAZ: >16 µg/mL as 32 µg/mL; FEP: ≤1 or >16 µg/mL as 1 or 32 µg/mL; C/T: ≤1/4 µg/mL as 1/4 µg/mL; ATM: ≤0.5 or >16 µg/mL as 0.5 or 32 µg/mL; IPM: ≤0.25 or >8 µg/mL as 0.25 or 16 µg/mL; and MEM: ≤0.125 or >8 µg/mL as 0.125 or 16 µg/mL. PIP, piperacillin; TZP, piperacillin/tazobactam; CAZ, ceftazidime; FEP, cefepime; C/T, ceftolozane/tazobactam; FDC, cefiderocol; ATM, aztreonam; IPM, imipenem; MEM, meropenem.

A total of 97 isolates were included in this study, none of which harbored carbapenemase and ESBL genes evaluated in this study. The MIC profiles of the isolates are shown in Table 1 and Fig. 1, while annual trends in antimicrobial susceptibility are presented in Table S2. The susceptibility rates to piperacillin, piperacillin/tazobactam, ceftazidime, and cefepime were in the 80% range, while 74.2% and 80.4% were susceptible to imipenem and meropenem, respectively. No isolate showed resistance to ceftolozane/tazobactam or cefiderocol, and only one isolate each showed intermediate resistance (susceptibility rate, 99.0% each). Among the β-lactams evaluated in this study, the lowest susceptibility rate was observed for aztreonam (71.1%). Detailed susceptibility patterns are provided in Table S3. The most prevalent susceptibility pattern was susceptible to all agents (56 isolates), followed by not susceptible only to aztreonam (nine isolates) or imipenem (seven isolates), and susceptible only to both ceftolozane/tazobactam and cefiderocol (six isolates).

**TABLE 1:** Antimicrobial susceptibility profiles of 97 *Pseudomonas aeruginosa* isolates

Antimicrobial agent	Minimum inhibitory concentration (µg/mL)			Susceptibility [% (number)]		
	Range	50%	90%	Susceptible	Intermediate	Resistant
Piperacillin	≤2–>64	4	32	86.6 (84)	5.2 (5)	8.2 (8)
Piperacillin/tazobactam	≤4/4–>64/4	≤4/4	32/4	87.6 (85)	4.1 (4)	8.2 (8)
Ceftazidime	1–>16	2	16	89.7 (87)	2.1 (2)	8.2 (8)
Cefepime	≤1–>16	4	16	86.6 (84)	8.2 (8)	5.2 (5)
Ceftolozane/tazobactam	≤1/4–8/4	≤1/4	≤1/4	99.0 (96)	1.0 (1)	0.0 (0)
Cefiderocol	≤0.06–8	0.125	0.5	99.0 (96)	1.0 (1)	0.0 (0)
Aztreonam	≤0.5–>16	8	>16	71.1 (69)	9.3 (9)	19.6 (19)
Imipenem	≤0.25–>8	2	>8	74.2 (72)	8.2 (8)	17.5 (17)
Meropenem	≤0.125–>8	0.5	>8	80.4 (78)	6.2 (6)	13.4 (13)

**Fig 2:**
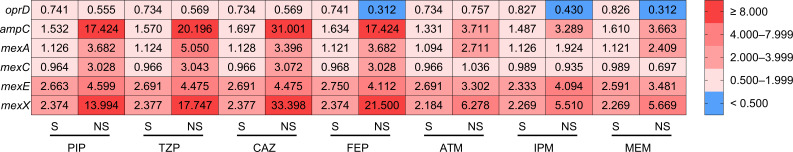
Heat map showing gene expression levels correlated with antimicrobial susceptibility profiles in 97 *Pseudomonas aeruginosa* isolates. Data are expressed as the median gene expression level for each antimicrobial agent. PIP, piperacillin; TZP, piperacillin/tazobactam; CAZ, ceftazidime; FEP, cefepime; ATM, aztreonam; IPM, imipenem; MEM, meropenem; S, susceptible; NS, not susceptible including intermediate and resistant.

**Fig 3:**
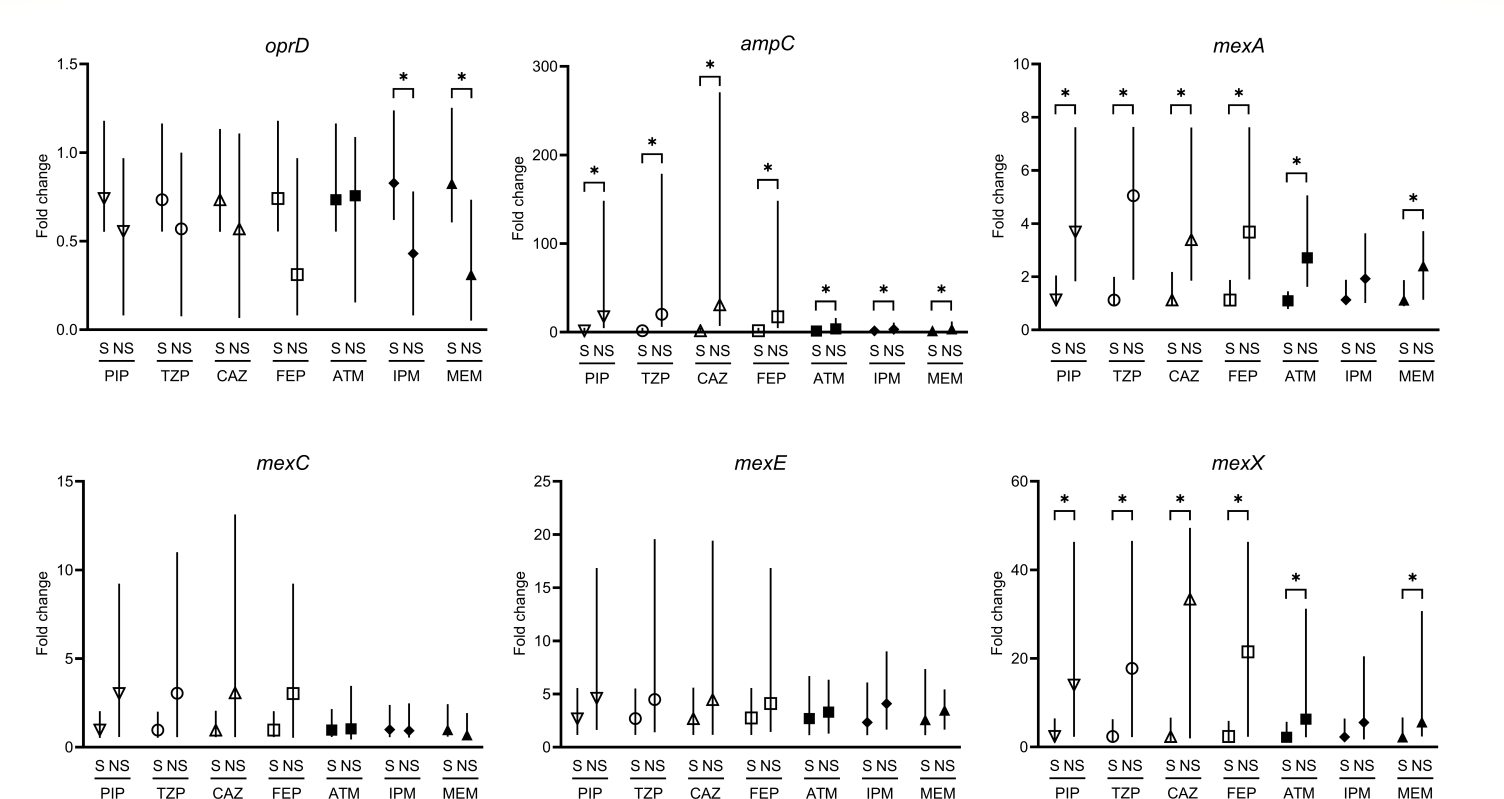
Comparison of resistance gene expression between susceptible and non-susceptible isolates in 97 *Pseudomonas aeruginosa* isolates. Data are expressed as median gene expression levels with interquartile ranges. * indicates statistical significance at *P* <0.05. PIP, piperacillin; TZP, piperacillin/tazobactam; CAZ, ceftazidime; FEP, cefepime; ATM, aztreonam; IPM, imipenem; MEM, meropenem; S, susceptible; NS, not susceptible including intermediate and resistant.

Differences in resistance gene expression between the susceptible and non-susceptible isolates are shown in Fig. 2 and 3 and Table S4. Significant increases in *ampC*, *mexA*, and *mexX* expression were commonly observed in non-susceptible isolates for piperacillin, piperacillin/tazobactam, ceftazidime, cefepime, and aztreonam. The decreased *oprD* and increased *ampC* expression was observed in both non-imipenem- and meropenem-susceptible isolates. In addition, *mexA* and *mexX* expression was significantly higher in non-meropenem-susceptible isolates.

**Fig 4:**
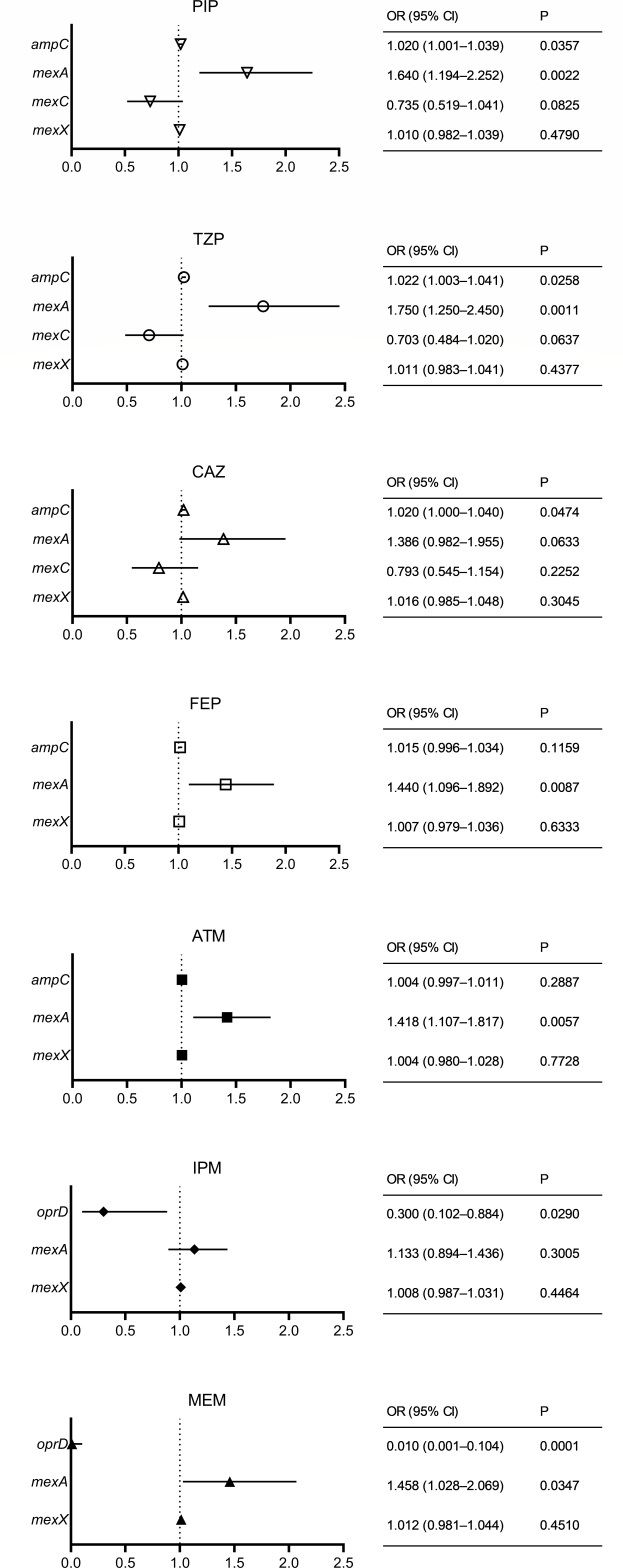
Multivariate analysis of the correlations between resistance gene expression and non-susceptibility (intermediate and resistant) in 97 *Pseudomonas aeruginosa* isolates. Data are expressed as odds ratios with corresponding 95% confidence intervals. PIP, piperacillin; TZP, piperacillin/tazobactam; CAZ, ceftazidime; FEP, cefepime; ATM, aztreonam; IPM, imipenem; MEM, meropenem; OR, odds ratio; CI, confidence interval.

The results of the univariate and multivariate analyses used to detect correlations between resistance gene expression and non-susceptibility (intermediate and resistant) to each β-lactam are shown in Table S5 and Fig. 4, respectively. In the univariate analysis, decreased *oprD* expression was found to be significantly correlated with decreased susceptibility to imipenem and meropenem. A significant correlation between increased *ampC* or *mexX* expression and non-susceptibility was observed in piperacillin, piperacillin/tazobactam, ceftazidime, and cefepime. Increased *mexA* expression was found to be significantly correlated with non-susceptibility to piperacillin, piperacillin/tazobactam, ceftazidime, cefepime, aztreonam, and meropenem. As a result of multivariate analysis, decreased *oprD* expression was found to be significantly correlated with decreased susceptibility to imipenem and meropenem. A significant correlation was observed between increased *ampC* expression and the non-susceptibility to piperacillin, piperacillin/tazobactam, and ceftazidime. Increased *mexA* expression was significantly correlated with decreased susceptibility to piperacillin, piperacillin/tazobactam, cefepime, aztreonam, and meropenem.

## DISCUSSION

In this study, antimicrobial susceptibility of *P. aeruginosa* isolates to major antipseudomonal β-lactams, including newer cephalosporins (e.g., ceftolozane/tazobactam and cefiderocol), was demonstrated. The results also highlighted the correlation between the expression of genes associated with resistance and a decreased susceptibility in *P. aeruginosa* isolates obtained from the blood in our hospital. The isolates evaluated in this study harbored no carbapenemase genes; however, their non-susceptibility rates to carbapenems ranged from 19.6% (meropenem, 19/97) to 25.8% (imipenem, 25/97) (Table 1). Decreased *oprD* expression was significantly correlated with non-susceptibility to imipenem and meropenem, whereas increased *mexA* expression was correlated with decreased susceptibility to meropenem (Fig. 4). These results are consistent with previous reports on *P. aeruginosa*, which showed that OprD loss is one of the most prevalent mechanisms related to carbapenem resistance, particularly to imipenem (11, 23). Additionally, the study found that the MICs for meropenem increased in isolates that overexpressed MexAB-OprM in combination with OprD loss (11). Furthermore, there is a potential for mutational variants of the chromosomally encoded AmpC enzyme to confer carbapenem resistance to *P. aeruginosa* (24, 25).

Among the 97 isolates evaluated, non-susceptibility to piperacillin, piperacillin/tazobactam, ceftazidime, and cefepime was observed in 10.3% (ceftazidime, 10/97) to 13.4% (piperacillin and cefepime, 13/97) (Table 1). Multivariate analyses showed that increased *ampC* and *mexA* expression was significantly correlated with non-susceptibility to piperacillin and piperacillin/tazobactam, and *ampC* or *mexA* expression was correlated with that to ceftazidime or cefepime (Fig. 4). Mutation-dependent overproduction of intrinsic AmpC is considered the main cause of the resistance of *P. aeruginosa* to antipseudomonal penicillins and cephalosporins (12). The susceptibility of the *P. aeruginosa* strain with *mexAB-oprM* deletion to piperacillin was found to be recovered compared to that of the wild type (13). The MexAB-OprM pump contributes to increased MICs for β-lactams, including cefepime, in both β-lactamase-negative and β-lactamase-inducible strains. However, the contribution is negligible if strains overexpress β-lactamase (26). The rate of non-susceptibility to aztreonam was 28.9% (28/97) (Table 1), and an increase in the *mexA* expression was correlated with a decreased susceptibility (Fig. 4). In a previous study, the hyperexpression of MexAB-OprM caused by a mutation in MexR was found to contribute to increased resistance to aztreonam (27).

In this study, only one isolate each showed intermediate resistance to ceftolozane/tazobactam and cefiderocol, and no isolate was resistant. In a previous study, the increased MIC related to AmpC β-lactamase was lower in ceftolozane compared with ceftazidime, indicating that ceftolozane is relatively stable against AmpC β-lactamase (28). However, recent studies have found that AmpC mutations can increase the MIC of ceftolozane/tazobactam (29, 30). In the present study, among the isolates evaluated, the highest level of *ampC* expression (551.328-fold) was observed in one non-ceftolozane/tazobactam-susceptible isolate (*ampC* expression in the remaining 96 susceptible isolates: 0.036–455.617-fold) (Table S4). Furthermore, the expression levels of the remaining genes related to resistance in the non-susceptible isolate were almost within the ranges of expression in the susceptible isolates. These results indicate that the overexpression of AmpC may cause an increase in the MIC of ceftolozane/tazobactam. In another study, no strong correlation was observed between the MIC of ceftolozane/tazobactam and OprD loss or hyperexpression in efflux systems (8). For cefiderocol, alterations in the target binding sites of *P. aeruginosa*-derived AmpC β-lactamases can reduce the activity of cefiderocol (30). When a transposon was inserted in either *mexR* or *nalD*, which leads to the overexpression of the MexAB-OprM efﬂux pump, the MIC increase due to a transposon insertion tended to be lower in ceﬁderocol than in ceftazidime and aztreonam. In addition, the MIC increase caused by transposon insertion in *oprD* was found to be lower in ceﬁderocol than in imipenem (31). In this study, the expression of all resistance genes in the non-cefiderocol-susceptible isolate was within the range of expression in the susceptible isolates. The increased MIC of cefiderocol might be conferred by other mechanisms, such as reduced influx caused by mutations in siderophore receptors of the iron transport system (32).

No carbapenemase producer was included in the study. The IMP-type metallo-β-lactamase, the main carbapenemase in *P. aeruginosa* in Japan, including in our hospital, is known to confer resistance to piperacillin, ceftazidime, and imipenem (14, 33). A recent nationwide surveillance study, which included 382 meropenem-resistant *P. aeruginosa* isolates collected from 78 Japanese hospitals between 2019 and 2020, revealed that only 4.2% (16/382) possessed carbapenemases (14 isolates with IMP type and 2 isolates with GES type) (34).

This study has some limitations. First, we could not assess patient-level clinical factors such as comorbidities, prior antibiotic exposure, and length of hospital stay. Further analysis, including these clinical factors as potential confounders, might enhance the clinical relevance and interpretability of our results. Second, while our logistic regression analyses revealed the correlations between non-susceptibility and resistance gene expressions, we could not determine the causes leading to changes in resistance gene expression. Further studies, such as whole-genome sequencing or targeted mutation analysis, are necessary to elucidate specific mutations responsible for changes in gene expression.

In conclusion, this study identified and characterized the correlations between the expression of genes encoding for AmpC β-lactamase, OprD porin, and efflux pumps and non-susceptibility to β-lactams in *P. aeruginosa* isolates from blood. These findings highlight the potential of ceftolozane/tazobactam and cefiderocol for use against *P. aeruginosa* isolates exhibiting antimicrobial resistance in BSI.

## SUPPLEMENTAL MATERIAL

Supplemental tables spectrum.02790-24-s0001.docx Tables S1 to S5.

ASM does not own the copyrights to Supplemental Material that may be linked to, or accessed through, an article. The authors have granted ASM a non-exclusive, world-wide license to publish the Supplemental Material files. Please contact the corresponding author directly for reuse.
