# Supplementary material for: Antimicrobial susceptibility and resistance mechanisms to antipseudomonal β-lactams in Pseudomonas aeruginosa isolates from blood
Source: Microbiol Spectr. 2025 Mar 31;13(5):e02790-24. doi: 10.1128/spectrum.02790-24 (PMC12054155; doi:10.1128/spectrum.02790-24)
Supplement: Supplemental tables — Tables S1 to S5. [file spectrum.02790-24-s0001.docx]

Supplementary Table 1. Primer sequences used for gene expression analysis

| Target | Primer | Sequence | Reference |
| --- | --- | --- | --- |
| *oprD*  *ampC*  *mexA*  *mexC*  *mexE*  *mexX*  *16S rRNA* | Forward  Reverse  Forward  Reverse  Forward  Reverse  Forward  Reverse  Forward  Reverse  Forward  Reverse  Forward  Reverse | 5′-CGGCGACATCAGCAACACC-3′  5′-GGGCCGTTGAAGTCGGAGTA-3′  5′-GGTGCAGAAGGACCAGGCACAGAT-3′  5′-CGATGCTCGGGTTGGAATAGAGGC-3′  5′-GGCGACAACGCGGCGAAGG-3′  5′-CCTTCTGCTTGACGCCTTCCTGC-3′  5′-GCAATAGGAAGGATCGGGGCGTTGG-3′  5′-CCTCCACCGGCAACACCATTTCG-3′  5′-TCATCCCACTTCTCCTGGCGCTACC-3′  5′-CGTCCCACTCGTTCAGCGGTTGTTCGATG-3′  5′-AATCGAGGGACACCCATGCACATCC-3′  5′-CCCAGCAGGAATAGGGCGACCAG-3′  5′-CCTACGGGAGGCAGCAG-3′  5′-ATTACCGCGGCTGCTGG-3′ | (16)  (16)  (16)  (16)  (16)  (16)  (16)  (16)  (16)  (16)  (16)  (16)  (17, 18)  (17, 18) |

Supplementary Table 2. Annual trends in antimicrobial susceptibility rates of 97 *Pseudomonas aeruginosa* isolates

|  | 2013 (n = 10) | 2014 (n = 6) | 2015 (n = 7) | 2016 (n = 11) | 2017 (n = 18) | 2018 (n = 19) | 2019 (n = 16) | 2020 (n = 10) |
| --- | --- | --- | --- | --- | --- | --- | --- | --- |
| Piperacillin  Piperacillin/tazobactam  Ceftazidime  Cefepime  Ceftolozane/tazobactam  Cefiderocol  Aztreonam  Imipenem  Meropenem | 80.0 (8)  80.0 (8)  80.0 (8)  80.0 (8)  100.0 (10)  100.0 (10)  50.0 (5)  70.0 (7)  90.0 (9) | 83.3 (5)  83.3 (5)  100.0 (6)  83.3 (5)  100.0 (6)  100.0 (6)  66.7 (4)  66.7 (4)  66.7 (4) | 85.7 (6)  85.7 (6)  85.7 (6)  85.7 (6)  100.0 (7)  100.0 (7)  85.7 (6)  71.4 (5)  85.7 (6) | 100.0 (11)  100.0 (11)  100.0 (11)  100.0 (11)  100.0 (11)  90.9 (10)  90.9 (10)  81.8 (9)  90.9 (10) | 77.8 (14)  83.3 (15)  83.3 (15)  83.3 (15)  94.4 (17)  100.0 (18)  72.2 (13)  66.7 (12)  77.8 (14) | 84.2 (16)  84.2 (16)  89.5 (17)  84.2 (16)  100.0 (19)  100.0 (19)  68.4 (13)  89.5 (17)  84.2 (16) | 87.5 (14)  87.5 (14)  87.5 (14)  81.3 (13)  100.0 (16)  100.0 (16)  75.0 (12)  75.0 (12)  68.8 (11) | 100.0 (10)  100.0 (10)  100.0 (10)  100.0 (10)  100.0 (10)  100.0 (10)  60.0 (6)  60.0 (6)  80.0 (8) |

Data are expressed as % (number).

Supplementary Table 3. Antimicrobial susceptibility patterns of 97 *Pseudomonas aeruginosa* isolates to β-lactam

| Antimicrobial | Susceptibility pattern | | | | | | | | | | | | | | | |
| --- | --- | --- | --- | --- | --- | --- | --- | --- | --- | --- | --- | --- | --- | --- | --- | --- |
| Piperacillin  Piperacillin/tazobactam  Ceftazidime  Cefepime  Ceftolozane/tazobactam  Cefiderocol  Aztreonam  Imipenem  Meropenem  Number | S  S  S  S  S  S  S  S  S  56 | S  S  S  S  S  S  S  NS  S  7 | S  S  S  S  S  S  S  NS  NS  3 | S  S  S  S  S  S  NS  S  S  9 | S  S  S  S  S  S  NS  S  NS  2 | S  S  S  S  S  S  NS  NS  S  1 | S  S  S  S  S  S  NS  NS  NS  4 | S  S  S  S  S  NS  S  S  S  1 | S  S  S  NS  S  S  NS  NS  NS  1 | NS  S  S  S  S  S  S  NS  NS  1 | NS  NS  S  NS  S  S  NS  S  S  1 | NS  NS  S  NS  S  S  NS  NS  NS  1 | NS  NS  NS  NS  S  S  S  NS  NS  1 | NS  NS  NS  NS  S  S  NS  S  S  2 | NS  NS  NS  NS  S  S  NS  NS  NS  6 | NS  NS  NS  NS  NS  S  NS  S  S  1 |

S, susceptible; NS, not susceptible including intermediate and resistant.

Supplementary Table 4. Comparison of resistance gene expression between susceptible and non-susceptible strains in 97 *Pseudomonas aeruginosa* isolates

| Variables | Susceptible | Non-susceptible (intermediate and resistant) | P |
| --- | --- | --- | --- |
| Piperacillin  *oprD*  *ampC*  *mexA*  *mexC*  *mexE*  *mexX*  Piperacillin/tazobactam  *oprD*  *ampC*  *mexA*  *mexC*  *mexE*  *mexX*  Ceftazidime  *oprD*  *ampC*  *mexA*  *mexC*  *mexE*  *mexX*  Cefepime  *oprD*  *ampC*  *mexA*  *mexC*  *mexE*  *mexX*  Ceftolozane/tazobactam  *oprD*  *ampC*  *mexA*  *mexC*  *mexE*  *mexX*  Cefiderocol  *oprD*  *ampC*  *mexA*  *mexC*  *mexE*  *mexX*  Aztreonam  *oprD*  *ampC*  *mexA*  *mexC*  *mexE*  *mexX*  Imipenem  *oprD*  *ampC*  *mexA*  *mexC*  *mexE*  *mexX*  Meropenem  *oprD*  *ampC*  *mexA*  *mexC*  *mexE*  *mexX* | n = 84  0.741 (0.553–1.180, 0.028–4.852)  1.532 (0.646–4.964, 0.036–138.302)  1.126 (0.909–2.049, 0.077–11.364)  0.964 (0.521–2.033, 0.192–43.559)  2.663 (1.142–5.559, 0.167–138.417)  2.374 (1.367–6.473, 0.419–153.572)  n = 85  0.734 (0.554–1.164, 0.028–4.852)  1.570 (0.650–4.958, 0.036–138.302)  1.124 (0.923–1.994, 0.077–11.364)  0.966 (0.536–2.007, 0.192–43.559)  2.691 (1.146–5.524, 0.167–138.417)  2.377 (1.370–6.294, 0.419–153.572)  n = 87  0.734 (0.553–1.133, 0.028–4.852)  1.697 (0.659–4.969, 0.036–138.302)  1.128 (0.951–2.177, 0.077–11.364)  0.966 (0.564–2.060, 0.192–43.559)  2.691 (1.154–5.594, 0.167–138.417)  2.377 (1.374–6.651, 0.419–153.572)  n = 84  0.741 (0.555–1.180, 0.028–4.852)  1.634 (0.661–4.964, 0.036–138.302)  1.121 (0.909–1.877, 0.077–11.364)  0.968 (0.570–2.033, 0.234–43.559)  2.750 (1.142–5.559, 0.167–138.417)  2.374 (1.367–5.890, 0.419–153.572)  n = 96  0.721 (0.473–1.099, 0.028–4.852)  1.999 (0.717–6.163, 0.036–455.617)  1.170 (0.964–2.399, 0.077–11.364)  0.968 (0.564–2.285, 0.192–43.559)  2.863 (1.184–6.503, 0.167–138.417)  2.550 (1.443–7.591, 0.419–153.572)  n = 96  0.741 (0.473–1.126, 0.028–4.852)  2.017 (0.728–6.294, 0.036–551.328)  1.170 (0.964–2.399, 0.077–11.364)  0.989 (0.570–2.386, 0.192–43.559)  2.863 (1.167–6.503, 0.167–138.417)  2.550 (1.391–7.591, 0.096–153.572)  n = 69  0.734 (0.554–1.164, 0.051–4.658)  1.331 (0.561–5.025, 0.036–209.246)  1.094 (0.787–1.453, 0.077–11.364)  0.966 (0.576–2.148, 0.234–43.559)  2.691 (1.138–6.693, 0.167–138.417)  2.184 (1.289–5.689, 0.419–153.572)  n = 72  0.827 (0.620–1.238, 0.177–4.852)  1.487 (0.597–5.455, 0.036–551.328)  1.126 (0.953–1.882, 0.126–11.364)  0.989 (0.564–2.386, 0.234–43.559)  2.333 (1.138–6.080, 0.167–71.801)  2.269 (1.367–6.425, 0.096–153.572)  n = 78  0.826 (0.606–1.253, 0.177–4.852)  1.610 (0.569–5.245, 0.036–551.328)  1.121 (0.890–1.876, 0.077–11.364)  0.989 (0.595–2.426, 0.234–43.559)  2.591 (1.138–7.354, 0.167–138.417)  2.269 (1.360–6.680, 0.096–153.572) | n = 13  0.555 (0.081–0.968, 0.051–3.797)  17.424 (4.504–148.311, 2.452–551.328)  3.682 (1.828–7.624, 0.211–8.511)  3.028 (0.577–9.226, 0.393–14.507)  4.599 (1.620–16.851, 0.666–66.744)  13.994 (2.294–46.321, 0.096–151.142)  n = 12  0.569 (0.076–1.000, 0.051–3.797)  20.196 (5.921–178.779, 2.452–551.328)  5.050 (1.886–7.637, 0.211–8.511)  3.043 (0.571–11.000, 0.393–14.507)  4.475 (1.414–19.562, 0.666–66.744)  17.747 (2.228–46.530, 0.096–151.142)  n = 10  0.569 (0.066–1.108, 0.051–3.797)  31.001 (7.071–270.839, 3.663–551.328)  3.396 (1.850–7.612, 0.211–7.907)  3.072 (0.566–13.137, 0.393–14.507)  4.475 (1.155–19.425, 0.666–66.744)  33.398 (1.940–49.481, 0.096–151.142)  n = 13  0.312 (0.081–0.968, 0.051–3.797)  17.424 (4.504–148.311, 0.641–551.328)  3.682 (1.898–7.624, 0.211–8.511)  3.028 (0.529–9.226, 0.192–14.507)  4.112 (1.430–16.851, 0.666–66.744)  21.500 (2.294–46.321, 0.096–151.142)  n = 1  3.797 (-, -)  551.328 (-, -)  1.873 (-, -)  14.507 (-, -)  1.209 (-, -)  0.096 (-, -)  n = 1  0.696 (-, -)  0.271 (-, -)  2.177 (-, -)  0.286 (-, -)  2.051 (-, -)  2.207 (-, -)  n = 28  0.757 (0.153–1.088, 0.028–4.852)  3.711 (1.758–16.077, 0.198–551.328)  2.711 (1.622–5.063, 0.152–8.511)  1.036 (0.436–3.459, 0.192–14.507)  3.302 (1.278–6.341, 0.666–66.744)  6.278 (2.205–31.245, 0.096–151.142)  n = 25  0.430 (0.081–0.781, 0.028–4.658)  3.289 (1.634–10.841, 0.057–209.246)  1.924 (1.012–3.634, 0.077–8.511)  0.935 (0.541–2.476, 0.192–7.625)  4.094 (1.657–9.010, 0.666–138.417)  5.510 (1.701–20.500, 0.419–151.142)  n = 19  0.312 (0.052–0.734, 0.028–1.219)  3.663 (1.697–12.038, 0.641–209.246)  2.409 (1.135–3.713, 0.211–8.511)  0.697 (0.393–1.924, 0.192–5.677)  3.481 (1.651–5.427, 0.666–22.273)  5.669 (2.370–30.719, 0.419–151.142) | 0.1313  < 0.001  0.0009  0.0933  0.2335  0.0134  0.1795  < 0.0001  0.0003  0.1184  0.2854  0.0143  0.2622  < 0.0001  0.0037  0.1079  0.4441  0.0185  0.0872  < 0.0001  0.0002  0.3170  0.3709  0.0054  -  -  -  -  -  -  -  -  -  -  -  -  0.3662  0.0057  < 0.0001  0.7053  0.5372  0.0047  0.0002  0.0207  0.1161  0.5721  0.2637  0.1033  < 0.0001  0.0100  0.0083  0.2249  0.6925  0.0431 |

Data are expressed as the median (interquartile range, absolute range) of the fold change, referred to the *P. aeruginosa* PAO1 strain after normalization using *16s rRNA*.

Supplementary Table 5. Univariate analysis of the correlations between resistance gene expression and non-susceptibility (intermediate and resistant) in 97 *Pseudomonas aeruginosa* isolates.

| Variables | Univariate analysis |  |
| --- | --- | --- |
|  | OR (95% CI) | P |
| Piperacillin  *oprD*  *ampC*  *mexA*  *mexC*  *mexE*  *mexX*  Piperacillin/tazobactam  *oprD*  *ampC*  *mexA*  *mexC*  *mexE*  *mexX*  Ceftazidime  *oprD*  *ampC*  *mexA*  *mexC*  *mexE*  *mexX*  Cefepime  *oprD*  *ampC*  *mexA*  *mexC*  *mexE*  *mexX*  Aztreonam  *oprD*  *ampC*  *mexA*  *mexC*  *mexE*  *mexX*  Imipenem  *oprD*  *ampC*  *mexA*  *mexC*  *mexE*  *mexX*  Meropenem  *oprD*  *ampC*  *mexA*  *mexC*  *mexE*  *mexX* | 0.683 (0.259–1.799)  1.026 (1.003–1.050)  1.509 (1.199–1.900)  1.060 (0.973–1.154)  1.010 (0.985–1.035)  1.028 (1.003–1.053)  0.724 (0.277–1.895)  1.028 (1.004–1.052)  1.563 (1.230–1.985)  1.063 (0.976–1.159)  1.011 (0.986–1.037)  1.030 (1.004–1.056)  0.835 (0.328–2.123)  1.029 (1.005–1.053)  1.399 (1.111–1.762)  1.071 (0.981–1.170)  1.011 (0.985–1.038)  1.032 (1.005–1.060)  0.622 (0.221–1.750)  1.026 (1.003–1.049)  1.545 (1.222–1.954)  1.057 (0.971–1.150)  1.010 (0.985–1.035)  1.033 (1.004–1.063)  1.038 (0.614–1.758)  1.007 (0.998–1.016)  1.472 (1.173–1.847)  1.026 (0.949–1.110)  1.000 (0.977–1.024)  1.023 (0.998–1.048)  0.345 (0.123–0.968)  0.999 (0.993–1.006)  1.136 (0.939–1.373)  0.921 (0.776–1.094)  1.011 (0.989–1.034)  1.013 (0.995–1.031)  0.021 (0.003–0.160)  1.000 (0.994–1.007)  1.221 (1.002–1.488)  0.850 (0.643–1.124)  0.974 (0.924–1.026)  1.016 (0.997–1.035) | 0.4402  0.0265  0.0005  0.1821  0.4287  0.0257  0.5106  0.0231  0.0003  0.1620  0.3770  0.0231  0.7048  0.0183  0.0043  0.1257  0.3931  0.0191  0.3685  0.0268  0.0003  0.2014  0.4539  0.0244  0.8882  0.1147  0.0009  0.5192  0.9956  0.0661  0.0433  0.8770  0.1887  0.3494  0.3288  0.1654  0.0002  0.9428  0.0477  0.2547  0.3166  0.0939 |

OR, odds ratio; CI, confidence interval.
